# Lectures on Diseases of the Skin, Delivered in the College of Medicine at Paris in 1841, 1842, 1843, 1844

**Published:** 1846-04

**Authors:** 


ART. XVI.

*Leçons sur les Maladies de la Peau, professées à l'Ecole de Médecine de Paris en* 1841, 1842, 1843, 1844. Par ALPH. CAZENAVE, Médecin de l'Hôpital Saint Louis, &c. Livraisons I, II, III.—*Paris,* 1845-6.

*Lectures on Diseases of the Skin delivered in the College of Medicine at Paris in* 1841, 1842, 1843, 1844. *Illustrated by coloured plates.* By ALPH. CAZENAVE, Physician to the Hospital of Saint Louis, &c. Parts I, II, III.—*Paris,* 1845-6. Folio.

WE hail with real pleasure the appearance of the work whose title heads this notice, and which is now in course of publication at Paris. The specimens before us are in every way worthy of the distinguished name which M. Cazenave has acquired as a dermatologist, and do honour to the celebrated institution—the Hospital of Saint Louis—to which the author has been attached, in the capacities of student and physician, for more than twenty years. The illustrations are extremely beautiful as works of art, and are faithful transcripts of the diseases which they are intended to represent. We have had an opportunity of comparing some of these plates with the actual cases, in the Hospital, from which they were drawn, and were gratified with the fidelity of the copies.

M. Cazenave proposes to complete his work in twelve Parts, one to appear every other month, and each Part to contain five coloured plates, folio, with about twenty pages of text, at the moderate price of 10s. It is sufficient for the present merely to direct our readers' attention to the work, reserving for a future period, when it shall be complete, a more extended examination of its contents. Before dismissing it, however, we may state briefly the plan the author proposes to follow. M. Cazenave has taught for several years, both at the Ecole de Médecine and at the Hospital of St. Louis, the necessity of viewing the subject of cutaneous pathology in a more philosophical light than has hitherto been done by those who have studied and practised that branch of medicine. It is not sufficient to know the *character* of an eruption,—to be able to tell whether it belongs to the vesicular, papular, or pustular groups. Although accuracy of diagnosis and a precise nomenclature are elements of the first importance in the study of this class of diseases, still they are not (although the contrary has been too generally believed) the only requisites necessary for forming correct principles of treatment. We must endeavour to disclose the intimate nature of the diseases themselves—as far as they can be ascertained—and thereby arrive at a knowledge of those general laws which regulate their progress and duration. Microscopical anatomy, by giving us more precise views of the structure of the skin, has materially assisted in dispelling the absurd and empirical notions which have so long degraded this branch of medicine. It has taught us to take a more enlarged view of the subject,—to regard it as an important and integral part of general pathology; and it is through the same medium that we must hope to establish lasting principles on which to found an enlightened and rational method of cure.

As a means of diagnosis, M. Cazenave considers the classification of Willan as modified by Biett, and which the reader will remember is founded on the external character of the disease, to answer all purposes; but here its utility ceases. M. Cazenave himself adopts the following classification, as being more comprehensive and more in accordance with the present state of medical science; but, although he has taught it for several years, he does not put it forth as definitive and complete, for it is impossible to form a lasting classification of cutaneous diseases in the present progressive state of anatomical science.

First Group. *Inflammatory diseases.*

Order I. Non-specific eruptions which may assume an acute or chronic character.

Erythema, Erysipelas, Urticaria, Strophulus, Herpes, Eczema, Pemphigus, Impetigo, Ecthyma, Sycosis.

Order II. Non-specific eruptions which always assume a chronic character.

Rupia, Lepra, Psoriasis, Pytiriasis, Pellagra.

Order III. Acute specific eruptions.

Roseola, Rubeola, Scarlatina, Variola, Vaccinia, Varicella, Miliaria.

Order IV. Chronic specific eruptions.

Syphilides.

Second Group. *Diseases of the secretory apparatus.*

Order I. Lesions of the follicular secretion.

Acne, Porrigo favosa.

Order II. Lesions of the epidermis.

Icthyosis, Horny productions.

Order III. Lesions of the colouring matter.

*Loss of colour:* Albinismus, Vitiligo. *Changes of colour:* Slate-coloured skin, Ephilides, Nævi.

Third Group. *Hypertrophic diseases.*

Abnormal development of the diseased parts.

Elephantiasis Arabum, Molluscum, Frambœsia, Vernea, Nævi vasculares.

Fourth Group. *Destructive diseases.*

Destructive tendency in the parts affected.

Elephantiasis Grecorum, Aleppo Evil, Cheloidea, Lupus, Cancer.

Fifth Group. *Hemorrhagic diseases.*

Tendency of the blood to exude from the vessels of the skin: hemorrhagic diseases properly so called.

Purpura, Melanosis. (?)

Sixth Group. *Exalted sensibility of the skin.*

General or local Hyperthesia.

Lichen, Prurigo, Anesthesia.

Seventh Group. *Foreign bodies.*

Acarus, Pediculus, Pulex.

Eighth Group.

*Diseases of the hair:* Alopecia, Canitia, Plica.

*Diseases of the nails:* Onyxia.

